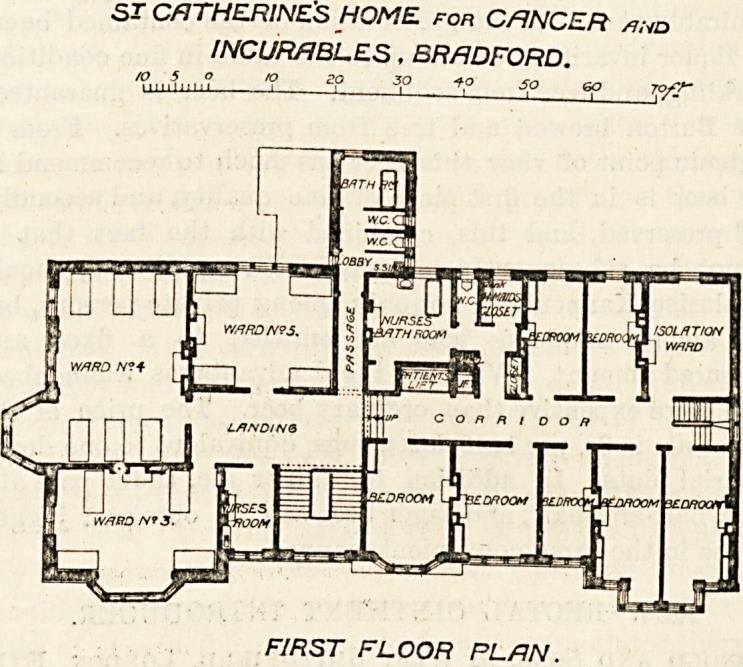# St. Catherine's Home for Cancer and Incurable Diseases, Bradford, Yorkshire

**Published:** 1901-05-11

**Authors:** 


### The Institutional Workshop.

### ST. CATHERINE'S HOME FOR CANCER AND INCURABLE DISEASES, BRADFORD, YORKSHIRE.

The Borough of Bradford owes this hospital entirely to the munificence of Mr. and Mrs. Cawthra, of Horton Hall, who have expended a sum of £10,000 on the structure, and who, therefore, deserve not a little praise for carrying out a work as much needed in the past as it will be appreciated in the future.

On examining the ground-floor plan it will be seen that the building is roughly divided into two sections by a porch, vestibule, outer hall, lift, and dispensary, all these being in line. On the right of the hall are the matron's room, linen room, servants' hall, and storeroom. The matron's room is entered direct from the outer hall, and the other rooms named from a corrider which divides them from the serving room, kitchen, larder, and scullery. At the end of the corridor is a door leading to a court in which the mortuary is placed, and which has a separate approach from the road. The section already described is the administrative part, and it is compact and well arranged. Turning to the left on passing through the outer hall we enter the inner hall, on the side of which is the principal staircase, the nurses' room, the day room, and a dormitory for six beds. Adjoining the latter is another dormitory for four beds. From this point the sanitary block projects. It is divided up the centre, and each division contains slop-sink, closet, and bath-room. The block is not cut off from the rest of the building by a cross-ventilating passage as it ought to have been, and cross-ventilation of the block itself must be impossible unless the divisional wall be stopped two or three feet from the ceiling. While the four-bedded dormitory has only a short lobby between it and the closet, the second division of the sanitary block is approached by a passage which can hardly fail to be insufficiently lighted.

ST CATHERINES HOME FOR CANCER AND INCURABLES. BRADFORD.

GROUND FLOOR PLAN.

The six-bedded dormitory is oblong in shape and has a fine bay window at one corner; it is only along this side of the room that other windows appear, hence cross ventilation cannot be properly carried out. The four-bedded room has only one window, in three sections, and part of it is shaded by the projecting bath-room.

The first floor plan is similar to the ground floor save that half of the sanitary block is not carried up, and that a nurses' bath and various bedrooms are provided over the administrative section already described, and that an isolation ward has been obtained over the scullery and larder.

A very successful attempt has been made to give the building a homely and non-institutional character both outside and inside; but we cannot help pointing out that this domestic character has been obtained at too great a sacrifice. Certainly the sanitary block ought to have been cut off from the main building in the usual way, and the dormitories at all events should have had three sides free and windows in two opposite sides enabling cross ventilation to be carried out.

The entire accommodation is for twenty-four patients. The basement is used as a laundry.

The home is warmed by hot water pipes; but very properly open fireplaces are also used; and the rooms are all lighted by electricity. All the wards are connected by flues to an exhaust ventilator in the roof, and the windows are specially contrived to admit pure air. These will lessen some of the drawbacks we have pointed out in the construction, but

ST CATHERINES HOME FOR CANCER AND INCURABLES, BRADFORD.

FIRST FLOOR PLAN.

they cannot altogether do away with them. The floors of the hall, corridor, and kitchen department are laid in marble mosaic, and those of the day rooms and dormitories are of maple blocks.

Several inhabitants of Bradford have come forward to help with the furnishing of the hospital. Miss Craig and Mr. Jacob Moser have furnished two of the large wards. Mr. James Gordon has fitted up the day room, and Dr. Rabagliati and Mr. Frederick Illingworth have also helped in the good work. Miss Hadley-Scott was appointed matron. The elevation is a very pleasing one in the modified Queen Anne style.

The architect was Mr. James Ledingham, F.R.I.B.A. The contractors were Messrs. Moulson and Son.

## Figures and Tables

**Figure f1:**
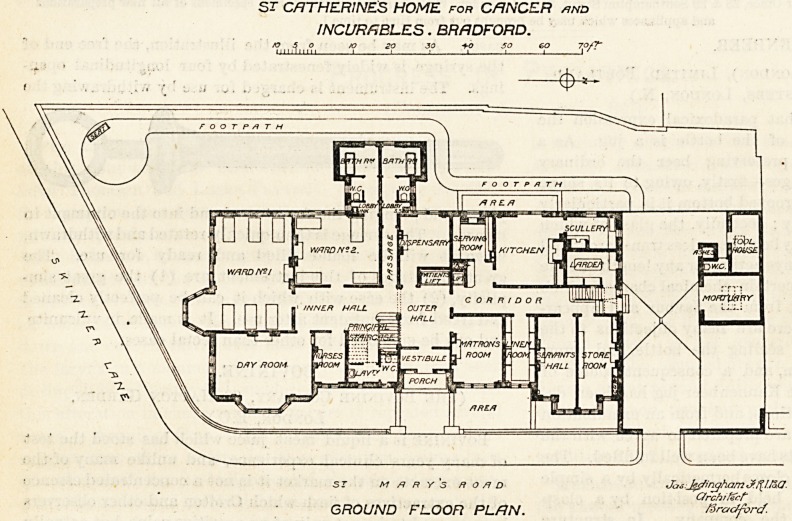


**Figure f2:**